# Quantitative-Profiling Method of Serum Steroid Hormones by Hydroxylamine-Derivatization HPLC–MS

**DOI:** 10.1007/s13659-019-0204-3

**Published:** 2019-04-09

**Authors:** Qi Liu, Quan Chi, Ru-Ting Fan, Hui-Dong Tian, Xian Wang

**Affiliations:** Key Laboratory of Analytical Chemistry of the State Ethnic Affairs Commission, College of Chemistry and Materials Science, South-Central University for Nationalities, Wuhan, 430074 Hubei People’s Republic of China

**Keywords:** Steroids, Derivatization, High performance liquid chromatography–mass spectrometry (HPLC–MS), Quantitative-profiling

## Abstract

**Abstract:**

A sensitive and rapid high performance liquid chromatography–mass spectrometry (HPLC–MS) method was developed and validated for simultaneous quantification of ten steroid hormones, including estrogens, androgens, progesterones, and corticosteroids four classes of steroids. The following ten steroid hormones were analyzed: progesterone, 21-deoxycortisol, estrone, 4-androstenedione, testosterone, dihydro-testosterone, androstenone, dehydroepiandrosterone, corticosterone and cortisone. Stable deuterated isotopes were used as internal standards for quantification. Sample preparation with and without derivatization were performed after liquid–liquid extraction, and the corresponding results were compared according to sensitivity and selectivity. Hydroxylamine derivatization was found to improve the ionization efficiency of the analytes for electrospray ionization MS analysis. The gradient of mobile phase and experimental parameters for HPLC separation were optimized. The lower limits of quantification were in the range of 0.05–5 ng mL^−1^ with wide linear range for the ten steroid hormones. The intra-day precision < 11.1% and recovery of 84.5–120% with negligible matrix effect were achieved, where within the acceptance limits of the FDA guideline. Total HPLC–MS analysis time was 6 min. This method enables simultaneous quantification of steroids in human serum. It will be helpful for the serum steroid profiling in order to understand various endocrinology diseases.

**Graphical Abstract:**

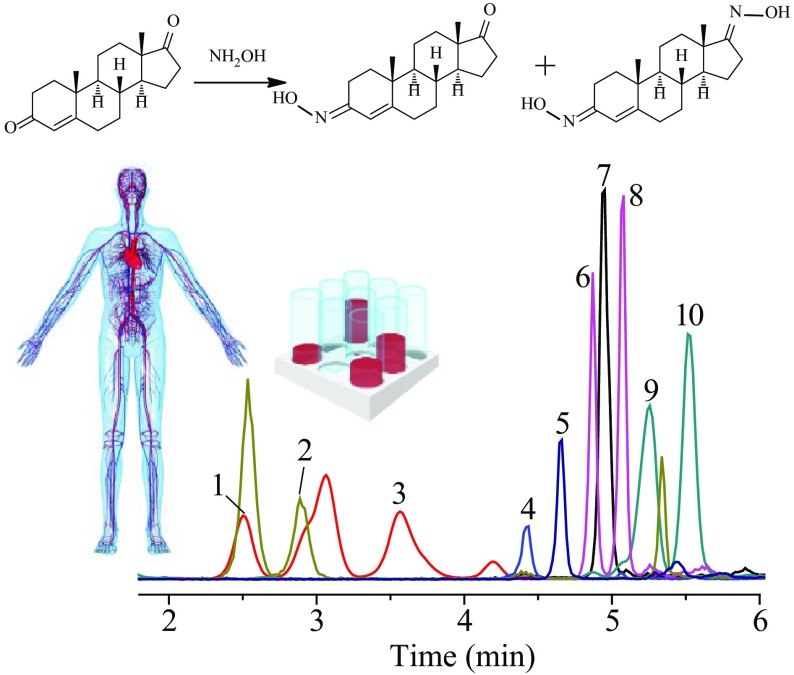

## Introduction

Steroid hormones are a class of tetracyclic aliphatic hydrocarbons with cyclopentane polyhydrophenanthrene nuclei, which play an important regulatory role in various physiological activities of human body and are indispensable hormones for maintaining life [1]. Steroids usually bind to nuclear receptors at a very low concentration of nano-molar or pico-molar levels. Because steroid hormones present in very low concentration in organisms, the structures of different steroid hormones are very similar and their polarity is relatively small, it is of challenge to detect and quantify steroids with high precision and accuracy.

Immunoassay is the mainstream method for steroid analysis in clinic in the past few decades, which has the advantages of high throughput and fast speed. However, this method is vulnerable to interference by body fluid matrix or structural analogues, resulting in the lack of specificity and accuracy [2, 3]. Therefore, gas chromatography mass spectrometry (GC–MS) was later used as the most accurate method [4, 5]. However, the analysis time of a GC–MS assay is often above 30 min, and its sensitivity is not as good as that of immunoassay [6]. Although GC–MS has a long history in steroid analysis, liquid chromatography-mass spectrometry (LC–MS) has become the current trend [7, 8]. LC–MS, LC–MS/MS methodology have been proved to have higher selectivity, sensitivity, throughput and simpler sample pretreatment process than radioimmunoassay, GC–MS and GC–MS/MS [8, 9].

The structural characteristics of steroid hormone lead to low ionization efficiency, which reduces sensitivity. In MS, ionization efficiency is closely related to hydroxyl, double bond and carbonyl groups contained in steroid hormone structure [10]. Some trace and non-ionizable hormones cannot be detected by conventional methods, but with the continuous improvement of ionization technology, chemical derivatization has become a mature strategy in improving the sensitivity and detection limit of steroid hormones [11, 12]. It not only improves the ionization efficiency during electrospray ionization (ESI) process by adding ionizable groups to the analyte which makes more analytes charged into mass spectrometry, but also changes the structure of the analyte and chromatographic separation behavior, and so more structural analogues or interferences can be separated.

There are various derivatization methods for steroid hormones. Estrogen can be treated with salmsulfonyl chloride or lipid derivatization [13, 14], whereas androgen can be derived with pyridinic acid [15, 16]. With LC–MS analysis, the detection limit of the above methods can reach < 10 pg mL^−1^. Other derivatives, such as alkylation, silylation and 2-hydrazinopyridination were detected by LC–MS with the lowest detection limit of 10 pg mL^−1^ [17–24]. Keski-Rahkonen et al. [19] used hydroxylamine hydrochloride solution as derivatization reagent after liquid–liquid extraction (LLE) and other some pretreatments, and the sensitivity of seven steroid hormones increased significantly. The hydroxylamine solution is the only derivative reagent that can simultaneously derive estrogens, androgens, corticosteroids and progesterones [25]. Although derivatization is shown to improve the ionization of analytes, it can also complicate the sample preparation process and uncontrollable reactions, such as geometric isomerization [15]. Therefore, it is worth considering and evaluating that whether or not derivatization has an advantage over non-derivatization method according to sensitivity, specificity and efficiency.

Aberrant changes of serum steroid hormones are closely associated with many endocrinological diseases, it is currently urgent need to develop a rapid and sensitive method for profiling serum steroid hormones by which we can considerably improve the diagnosis and therapeutics against the different conditions. In the present work, we aims at developing a rapid and sensitive method for simultaneous determination of ten steroid hormones, and to compare the difference in sensitivities for the pretreatments with and without the derivatization of hydroxylamine. The method has three advantages that are small sample consumption, short detection time and more hormones detected at one injection.

## Results and Discussion

### Chromatography

**Fig. 1:**
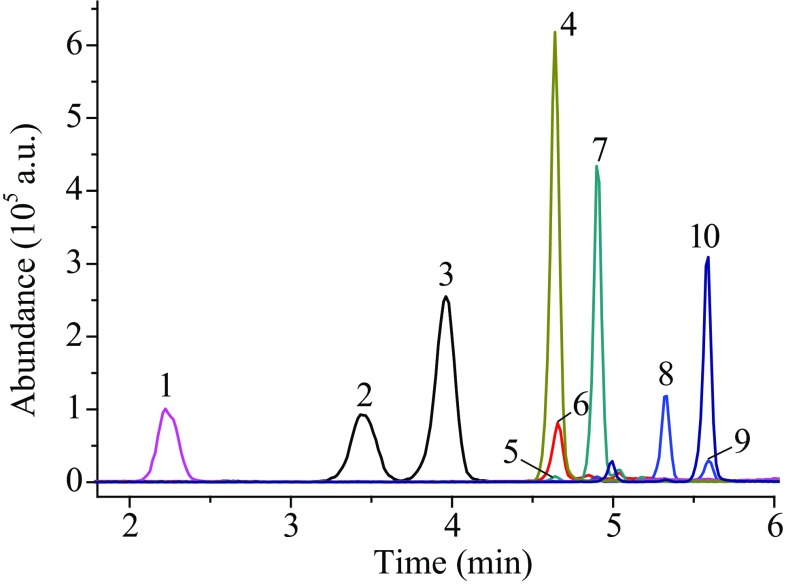
The extracted ion chromatogram (EIC) of ten steroids without derivatization at the concentration of 500 ng mL^−1^. 1—cortisone; 2—21-deoxycortisol; 3—corticosterone; 4—4-androstenedione; 5—dehydroepiandrosterone; 6—estrone; 7—testosterone; 8—dihydrotestosterone; 9—androsterone; 10—progesterone

**Fig. 2:**
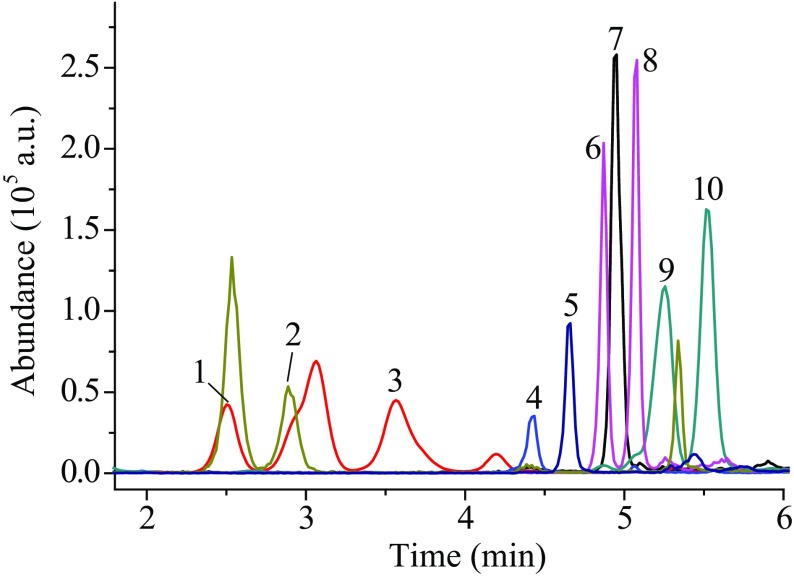
The extracted ion chromatogram (EIC) of ten steroid derivatives at the concentration of 50 ng mL^−1^. 1—21-deoxycortisol derivative; 2—cortisone derivative; 3—corticosterone derivative; 4—estrone derivative; 5—progesterone derivative; 6—dehydroepiandrosterone derivative; 7—4-androstenedione derivative; 8—testosterone derivative; 9—dihydrotestosterone derivative;10—androsterone derivative

Because of the advantages of improving separation ability, shortening analysis period and improving peak shape, gradient elution was used in this experiment. The mobile phase consisted of phase A (0.1% formic acid in methanol) and phase B (0.1% formic acid in water). Six gradients were optimized in gradient elution experiment. T_1_ (0.6 mL min^−1^), T_2_ (0.5 mL min^−1^) and T_3_ (0.4 mL min^−1^) were used to optimize the flow rate, and T_4_ (50% A), T_5_ (60% A) and T_6_ (70% A) were used to optimize the initial phase A ratio. The results showed that low flow rate could increase the separation degree of each hormones, but the retention time of each hormones was relatively prolonged. Different phase A ratio at the beginning of the gradients had a great impact on the separation degree. When the ratio of phase A was high, the separation degree of the early stage peak was smaller, and that of the later stage peak was larger. However, when its ratio was low, the separation degree of the peak in the early stage was larger, whereas the separation degree of the peak in the later stage was smaller and the total retention time was delayed. For the above reasons, the flow rate of 0.5 mL min^−1^ and the initial phase A ratio 60% were chosen as the optimized chromatographic condition. Under this condition, the steroid hormones were separated well, with little interference from other substances (Figs. 1, 2).

### Derivatization

**Fig. 3:**
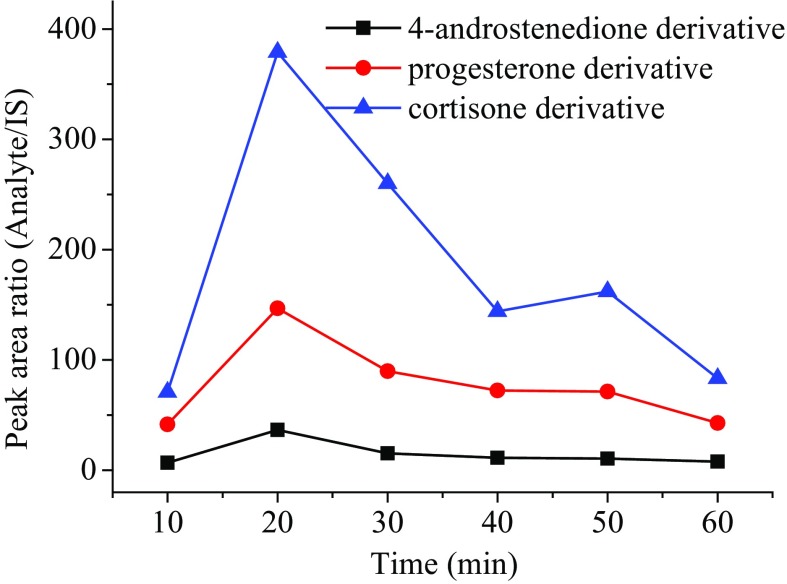
Derivative efficiencies of different reaction times for the representative steroids in human serum at reaction temperature of 40 °C

**Fig. 4:**
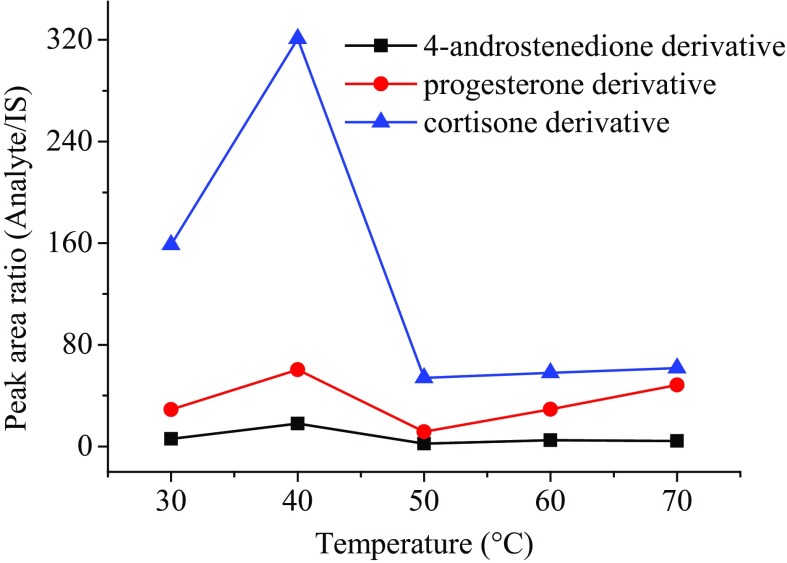
Derivative efficiencies of different reaction temperatures for the representative steroids in human serum at reaction time of 20 min

In order to optimize the reaction time of derivatization for hydroxylamine and steroid solutions, six reaction times (10 min, 20 min, 30 min, 40 min, 50 min and 60 min) at 40 °C were conducted and compared. Progesterone, 4-androstenedione and cortisone derivatives were chosen as the representatives to select the most suitable reaction time. The results in Fig. 3 shows that the reaction time of 20 min gives the maximum signal response in MS. Reaction temperatures were also optimized at 30 °C, 40 °C, 50 °C, 60 °C and 70 °C, respectively. As shown in Fig. 4, the reaction at 40 °C gives the highest derivatization efficiency.

### Comparison of the Sample Analysis With and Without Derivatization

**Fig. 5:**
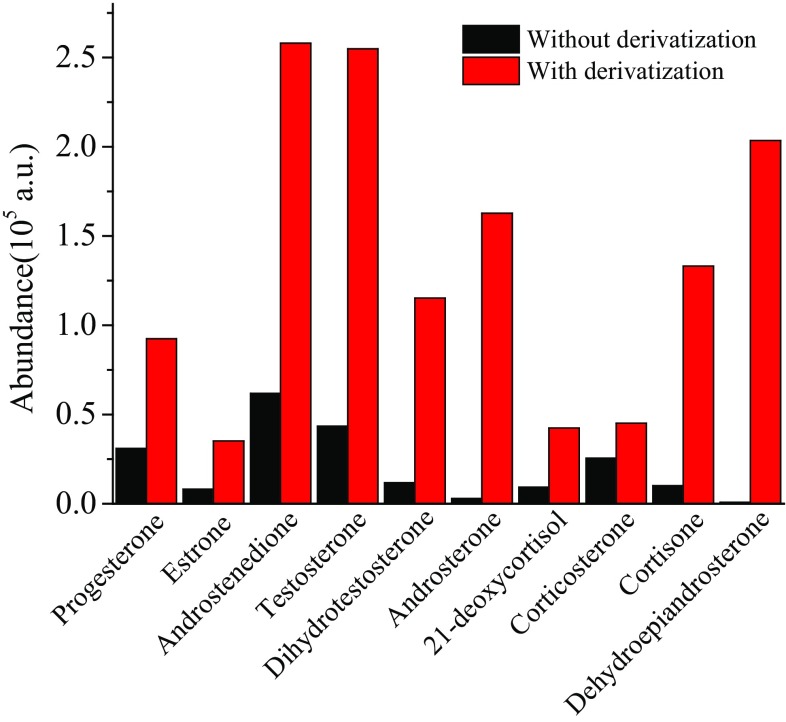
Comparison the ion intensity for the analysis with derivatization and without derivatization at the concentration of 50 ng mL^−1^

The analysis results of samples pretreated only by LLE were compared with those pretreated by LLE and derivatization. The extracted ion chromatogram (EIC) of ten steroids at the concentration of 500 ng mL^−1^ and their derivatives at the concentration of 50 ng mL^−1^ are given in Figs. 1 and 2, respectively. The results in Table 1 show that the quantitative limits of ten hormones have increased by 3.9–202.6 times with derivatization, and the corresponding signal intensities in MS of ten hormones have increased by about 1.8–251.9 times (Fig. 5). It indicates that hydroxylamine derivatization can significantly improve the ionization efficiency of the analytes at both low and high concentrations, and the signal responses of MS are greatly improved. From Table 1 the most significant enhancement of MS signal was observed for androsterone, dehydroepiandrosterone, 21-deoxycortisol and progesterone, at low concentrations. But at high concentrations (50 ng mL^−1^), the signal responses of androsterone and dehydroepiandrosterone increased significantly with derivatization, while 21-deoxycortisol and progesterone increased only 3 times and 4.6 times (Fig. 5). The reason may be that the side reaction of hydroxylamine derivatization increased at high concentrations, resulting in the decrease of main products and their mass spectrometry response.

**Table 1:** Comparison the LOQ for the analysis with derivatization and without derivatization

Analytes	No derivatization, LOQ (S/N ≥ 10, *n *= 3)			After derivatization, LOQ (S/N ≥ 10, *n *= 3)			Enhancement ratio
	ng mL^−1^	S/N	CV (%)	ng mL^−1^	S/N	CV (%)	
Progesterone	20	10.1	12.5	0.1	10.2	17.9	200
Estrone	21.1	10.5	14.3	1	10	11.6	21.1
Androstenedione	5	16.2	10.3	0.5	12.3	12.5	10.0
Testosterone	11.8	16.9	5.4	0.8	16.3	1.05	14.8
Dihydrotestosterone	25.4	13	8.9	1	13.9	15.1	25.4
Androsterone	101.3	15.2	9.8	0.5	12.6	6.9	202.6
21-Deoxycortisol	10	10.1	11.1	0.05	11.8	12.9	200.0
Corticosterone	5	10.2	9.2	1	12.4	1.8	5.0
Cortisone	19.5	15.9	6.4	5	13.3	1.5	3.9
Dehydroepiandrosterone	29.2	10	10.2	0.4	12.1	0.83	73.0

### Specificity and Selectivity

Three pairs of isomers and three pairs of distinct stereoisomers were obtained for the derivatization of ten steroid hormones with hydroxylamine. Among them, dihydrotestosterone derivatives and androsterone derivatives, testosterone derivatives and dehydroepiandrosterone derivatives, 21-deoxycortisol derivatives and corticosterone derivatives are the isomers. Progesterone derivatives and 21-deoxycortisol derivatives, corticosterone derivatives and cortisone derivatives have obvious stereo-isomerization phenomena. Under the optimal chromatographic conditions, the peak shape and separation effect were significant improved. The results showed that the method had good specificity. The selectivity of the method was evaluated by comparing chromatograms of blank plasma samples to spiked plasma samples at lower limit of detection (LOD). Some interferences were detected in the chromatograms, and the signal to noise ratio (S/N) of spiked to blank plasma at lower limit of quantitation (LOQ) was above 5, showing that this method had a good selectivity.

### Calibration Curve and Sensitivity

The LOQ was determined at S/N ≥ 10. Each sample at LOQ concentration was analyzed for three replicates, and the coefficient of variance (CV) was < 17.9%. The LOQ of this ten steroids was in the range of 0.05–5 ng mL^−1^. The calibration curves fitted well over the wide calibration range with regression correlation coefficient (R^2^) between 0.9895 and 0.9993 (Table 2).

**Table 2:** Calibration curve and LOQ for the ten steroid hormones

Analytes	Linear dynamic range (ng mL^−1^)	Regression line			LOQ (S/N ≥ 10, *n *= 3)		
		Slope	Intercept	R^2^	ng mL^−1^	S/N	CV (%)
Progesterone	0.1–100	0.0457	0.0128	0.9929	0.1	10.2	17.9
Estrone	1–100	0.0717	− 0.0674	0.9993	1	10	11.6
Androstenedione	0.5–100	0.0159	− 0.0181	0.997	0.5	12.3	12.5
Testosterone	1–100	0.0123	− 0.051	0.9946	0.8	16.3	1.05
Dihydrotestosterone	1–100	0.0116	− 0.029	0.9983	1	13.9	15.1
Androsterone	0.5–100	0.012	0.002	0.9969	0.5	12.6	6.9
21-Deoxycortisol	0.05–100	0.1806	− 0.0855	0.9985	0.05	11.8	12.9
Corticosterone	1–100	0.2662	− 0.1849	0.9992	1	12.4	1.8
Cortisone	5–100	0.3585	− 2.0132	0.9895	5	13.3	1.5
Dehydroepiandrosterone	0.5–100	0.0181	− 0.0452	0.999	0.4	12.1	0.83

### Precision, Accuracy and Matrix Effect

The results in Table 3 shows that the intra-day precision (RSD) of each steroids is < 11.1%, recovery ranges from 84.5 to 120%, and matrix effect is within 80.3–123.6%. These results demonstrate that the method has good precision, accuracy and little matrix effect, which meets the acceptance limits of the FDA guideline.

**Table 3:** Recovery, matrix effect and Intra-day precision

Analytes	Spiked amount (ng mL^−1^)	Intra-day precision (RSD, %)	Matrix effect (%)	Recovery (%)
Progesterone	5	1.4	86.9	96.3
	20	1.0	113.1	104.4
	100	4.9	107.5	102.4
Dehydroepiandrosterone	5	0.9	97.1	84.5
	20	0.5	105.7	97.8
	100	0.2	109.2	100.8
Testosterone	5	0.2	82.2	88.1
	20	5.1	85.3	87.3
	100	2.6	105.8	102.9
21-Deoxycortisol	5	5.0	120.7	104.8
	20	4.3	115.8	98.1
	100	1.4	107.6	101.6
Androstenedione	5	0.2	81.6	102.5
	20	4.0	83.5	94.0
	100	0.1	80.3	102.2
Androsterone	5	10.5	83.6	106.1
	20	10.6	82.3	93.9
	100	4.7	83.7	98.0
Dihydrotestosterone	5	0.4	83.5	114.5
	20	1.9	81.5	94.9
	100	0.9	80.7	101.2
Estrone	5	10.4	100.9	87.7
	20	11.1	100.1	101.7
	100	10.3	80.5	99.2
Cortisone	5	0.0	119.5	120.0
	20	0.1	120.0	91.1
	100	2.7	105.9	103.5
Corticosterone	5	0.0	109.7	99.4
	20	4.5	123.6	98.5
	100	2.7	102.1	100.9

## Experimental Section

### Chemicals and Solutions

Standard compounds, including progesterone, estrone, 4-androstenedione, testosterone, dihydrotestosterone, androsterone, 21-deoxycortisol, corticosterone, cortisone, dehydroepiandrosterone, progesterone-d9, testosterone-d3 and dehydropiandrosterone-d6, hydroxylamine solution and bovine serum albumin (BSA, fatty acid and globulin free, purity ≥ 99%) were purchased from Sigma-Aldrich (Shanghai, China). Formic acid was obtained from China Pharmaceutical Group (Beijing, China), and dexamethasone, methyl *tert*-butyl ether (MTBE) was from Aladdin (Shanghai, China). Phosphate buffered saline was obtained from Biological Industries (Shanghai, China). The water purification system was obtained from Mole Scientific Instrument Co. Ltd (Shanghai, China). HPLC-grade methanol was purchased from Fisher Scientific (Waltham, MA, USA). Other reagents were at least analytical grade and obtained commercially.

BSA solution was made by dissolving 2 g of BSA in 50 mL of phosphate buffered saline. For ten steroid hormones, stock standard solutions with the concentration of 5 × 10^−4^ g mL^−1^ in methanol were prepared, and a series of working standard solutions with the concentrations of 0.1–1000 ng mL^−1^ were prepared by further dilution with methanol. The internal standard (IS) solution was prepared by dissolving progesterone-d9, testosterone-d3, dehydropiandrosterone-d6 and dexamethasone in methanol and diluted progressively to the final concentration of 100 ng mL^−1^.

### Derivatization Reaction and Sample Preparation

**Fig. 6:**
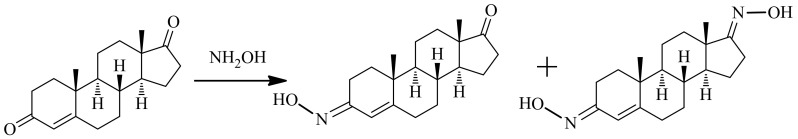
The derivatization reaction of 4-androstenedione with hydroxylamine

Serum samples were thawed at room temperature and protected from light. An aliquot of 180 μL was transferred into 2 mL screw top vial, spiked with 20 μL of IS solution and mixed shortly. Subsequently, 1 mL of MTBE was added into the solution. The vial was capped and shaken at 2000 rmp for 3 min. After the extraction, the organic layer was transferred into another vial, evaporated to dryness at 25 °C and then reconstituted in 100 μL hydroxylamine solution of 100 mmol L^−1^ (Fig. 6). The capped vial was heated at 40 °C for 20 min before placing into autosampler for analysis. Calibration samples were prepared similarly to the serum samples, only substituting the serum with 4% (w/v) BSA solution. In addition to the IS, 20 μL of working standard solutions were added into the vials before the extraction. The calibration curve consisted of thirteen concentration levels: 0.01 ng mL^−1^, 0.05 ng mL^−1^, 0.1 ng mL^−1^, 0.2 ng mL^−1^, 0.5 ng mL^−1^, 1 ng mL^−1^, 5 ng mL^−1^, 10 ng mL^−1^, 20 ng mL^−1^, 50 ng mL^−1^, 100 ng mL^−1^, a zero sample (only IS added), and a blank (no standards added). Quality control (QC) samples were prepared similarly to the calibration samples at three concentration levels: 5 ng mL^−1^, 20 ng mL^−1^, 100 ng mL^−1^, where within the acceptance limits of the FDA guideline.

### LC–MS Analysis

LC experiments were conducted on a model 1200 LC system (Agilent, Santa-Clara, CA, USA) with a Saifen GP-C18 (150 × 2.1 mm, 5 µm) with the column temperature of 40 °C, and the injection volume was 5 μL. The mobile phase consisted of eluent A (methanol with 0.1% formic acid) and eluent B (purified water with 0.1% formic acid) at a flow rate of 0.5 mL min^−1^. The solvent gradient was started at 60% A and held for 0.5 min, then programmed to 90% A in 1.5 min and held for another 1.5 min, and then returned to 60% A in 1.5 min and held for 1 min. The total run time for one injection was 6 min.

MS and MS/MS analysis were conducted on a model 6520 Q-TOF mass spectrometer (Agilent, Santa Clara, CA, USA) with a standard ESI source in the positive ion mode. The parameters of ESI–MS analysis were optimized: capillary voltage 3.5 kV, fragmentor voltage 75 V, dry gas flow rate 10 L min^−1^, dry gas temperature 350 °C, nebulizer pressure 50 psig. The mass spectra were acquired in the range of *m*/*z* 200–600. Mass spectrometric parameters of the analytes were given in Table 4.

**Table 4:** Mass spectrometric parameters of the analytes

Analytes	Molecular formula	MW	[M + H]^+^	[M + *n*NH + H]^+^	RT (min)
Estrone	C_18_H_22_ O_2_	270.37	271.37	286.18	4.10
Androstenedione	C_19_H_26_O_2_	286.41	287.41	317.22	4.85
Testosterone	C_19_H_28_O_2_	288.42	289.42	304.22	4.90
Dehydroepiandrosterone	C_19_H_28_ O_2_	288.43	289.43	304.22	4.70
Dihydrotestosterone	C_19_H_30_O_2_	290.44	291.44	306.24	5.20
Androsterone	C_19_H_30_ O_2_	290.44	291.44	306.24	5.50
Progesterone	C_21_H_20_O_2_	314.46	315.46	330.24	4.45
21-Deoxycortisol	C_21_H_30_ O_4_	346.46	347.46	377.24	2.30
Corticosterone	C_21_H_30_ O_4_	346.46	347.46	377.24	3.30
Cortisone	C_21_H_28_ O_5_	360.45	361.45	406.23	2.49

### Assay Validation

Assay validation was carried out according to the FDA guideline for bioanalytical method validation guidance for industry (May 2018). Assay validation was performed with respect to specificity, selectivity, linearity, LOD, LOQ, accuracy, precision and matrix effect, with the emphasis on the requirements for the analysis of endogenous compounds [26].

#### Specificity and Selectivity

The selectivity of the method was carried out by comparing the chromatogram of a blank plasma sample with spiked plasma sample at the LOD. Analytes response should be at least five times than that of the blank plasma and the method should distinguish the analyte from endogenous material or other metabolites, isomers and etc. The presence of isomers in the substance can easily lead to cross-reactions, so it is necessary to separate the substance on chromatography.

#### Linearity of Calibration Curve, LOD and LOQ

Calibration curves were created by plotting the peak areas versus the known concentrations of the calibration standards. The linearity of all analytes determined in spiked steroid free plasma were obtained for eleven calibration standards in three independent runs. Linear equation and correlation coefficient were recorded. Linearity was considered satisfactory when the correlation coefficients (R^2^) were above 0.99 over the concentration range. LOD is the lowest concentration of analyte that can be detected by mass spectrometry. LOD was evaluated by considering the lowest concentration at which the S/N was > 3. LOQ was determined as the concentration providing S/N of at least 10 within CV < 20%.

#### Precision and Accuracy

Three quality control solutions with different concentrations of 5 ng mL^−1^, 20 ng mL^−1^ and 100 ng mL^−1^ (low, medium and high) were selected to evaluate the intra-day precision and each concentrations was detected for three times. Because there is no reference method and reference material, the recovery experiment was used to evaluate the accuracy. Working solutions with known concentrations were added to three quality controls and each concentration was tested five times. The ratio of the measured value to the theoretical value was used to evaluate the accuracy [27].

#### Matrix Effect

The matrix effect of the method was studied by comparing the peak areas of the analytes of interest added into pre-extracted steroid free plasmas to the diluted standard solutions at the same concentrations using five different replicates. When the peak area ratio of analytes was between 80 and 120%, the matrix effect was considered to be negligible.

## Conclusions

In the current study, a sensitive method for the simultaneous determination of ten steroid hormones in human serum was established by LLE, derivatization, stable isotope labeling and HPLC–ESI–Q-TOF–MS. Chromatography and derivatization conditions were optimized to give the best separation and ionization efficiency in HPLC–MS. Although derivatization can causes the complexity of LC–MS analysis and is usually avoided, the sample preparation steps in this method was accomplished by using only two autosampler vials for extraction, derivatization and injection. In addition, significant increase in MS signal responses and sensitivities were obtained by employing oxime derivatization. Isotope labeling internal standards were used to ensure the accurate quantification. Assay validation was evaluated according to sensitivity, precision, matrix effect, recovery, selectivity and stability, where within the acceptance limits of the FDA guideline for bioanalytical method validation. The developed method can be further applied for the analysis of clinical human serum samples for steroids monitoring and endocrinology disease diagnosis.
